# Hepatoptosis by Interposition of Intestine

**Published:** 1918-11

**Authors:** 


**Hepatoptosis by Interposition of Intestine.** Laurent Moreau, Prog. Med. Mch. 16, describes two cases, demonstrated by X-rays, by means of the clear space between the liver and the diaphragm. While very rare, this condition has been described by Beclere, Aubourg and Perussia, the three important factors being abdominal hyperpressure, gaseous dilatation of the colon and malformation of the liver. (Note: Clinically, cases are encountered in which, without malformation of the liver, and with a question as to whether hyper or hypotension exists, coils of intestine, not necessarily the colon, come somewhat in front of and above the anterior portion of the dome of the liver.)

